# Supplementary material for: mTOR-Dependent Role of Sestrin2 in Regulating Tumor Progression of Human Endometrial Cancer
Source: Cancers (Basel). 2020 Sep 4;12(9):2515. doi: 10.3390/cancers12092515 (PMC7565818; doi:10.3390/cancers12092515)

Figure 1B

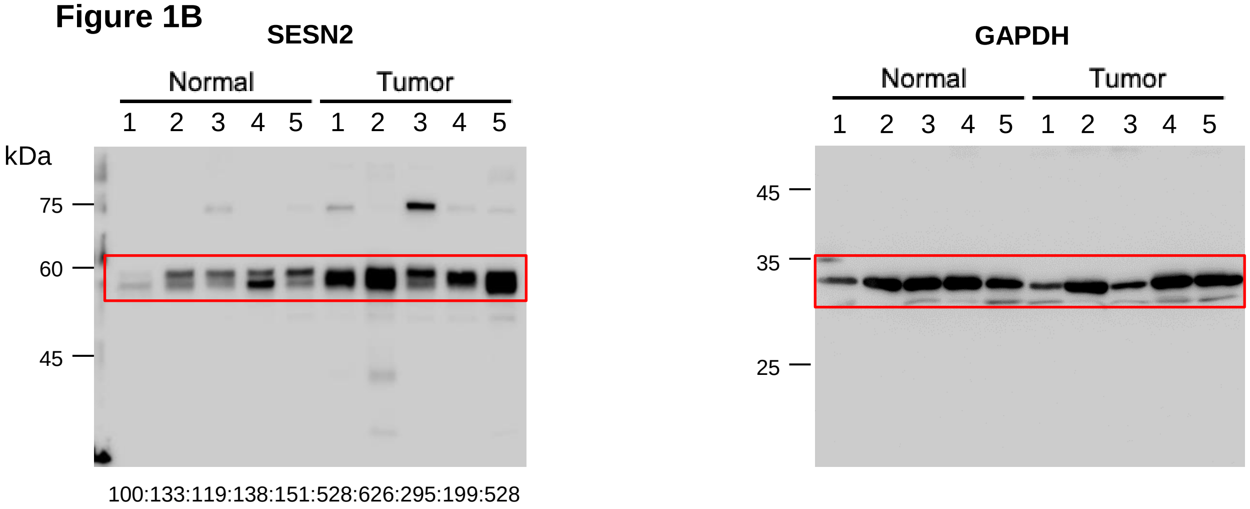

Figure 2A

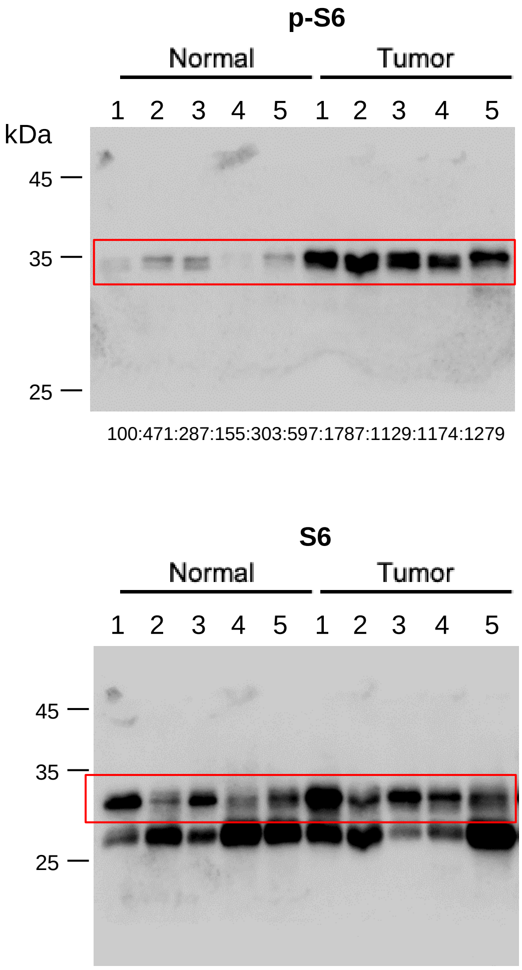

Figure 2E

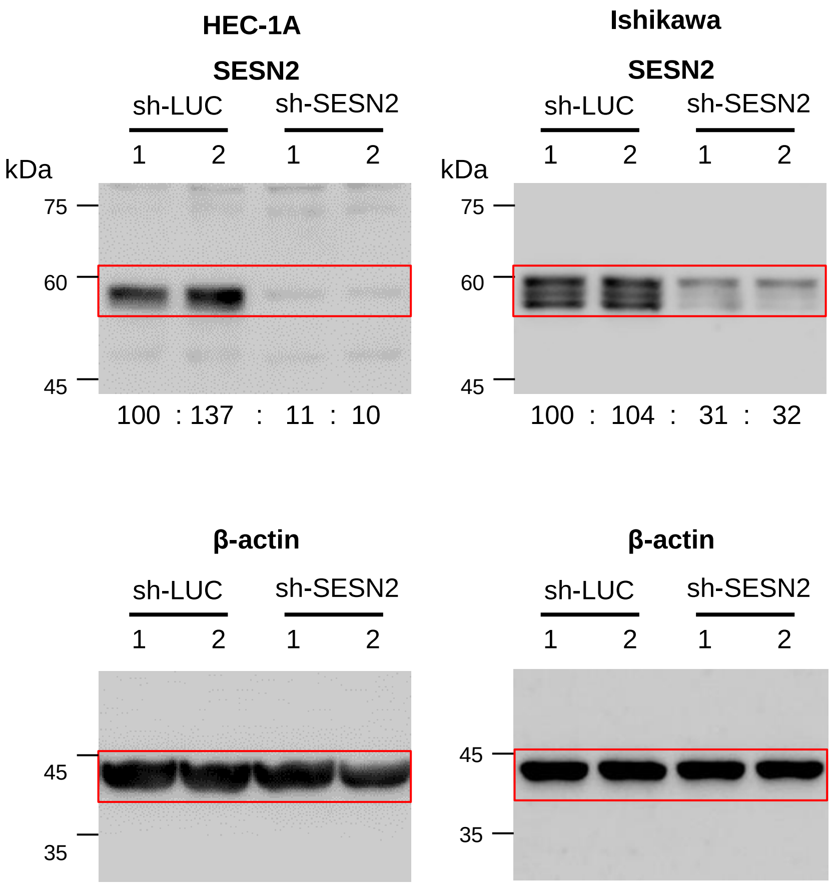

Figure 2F

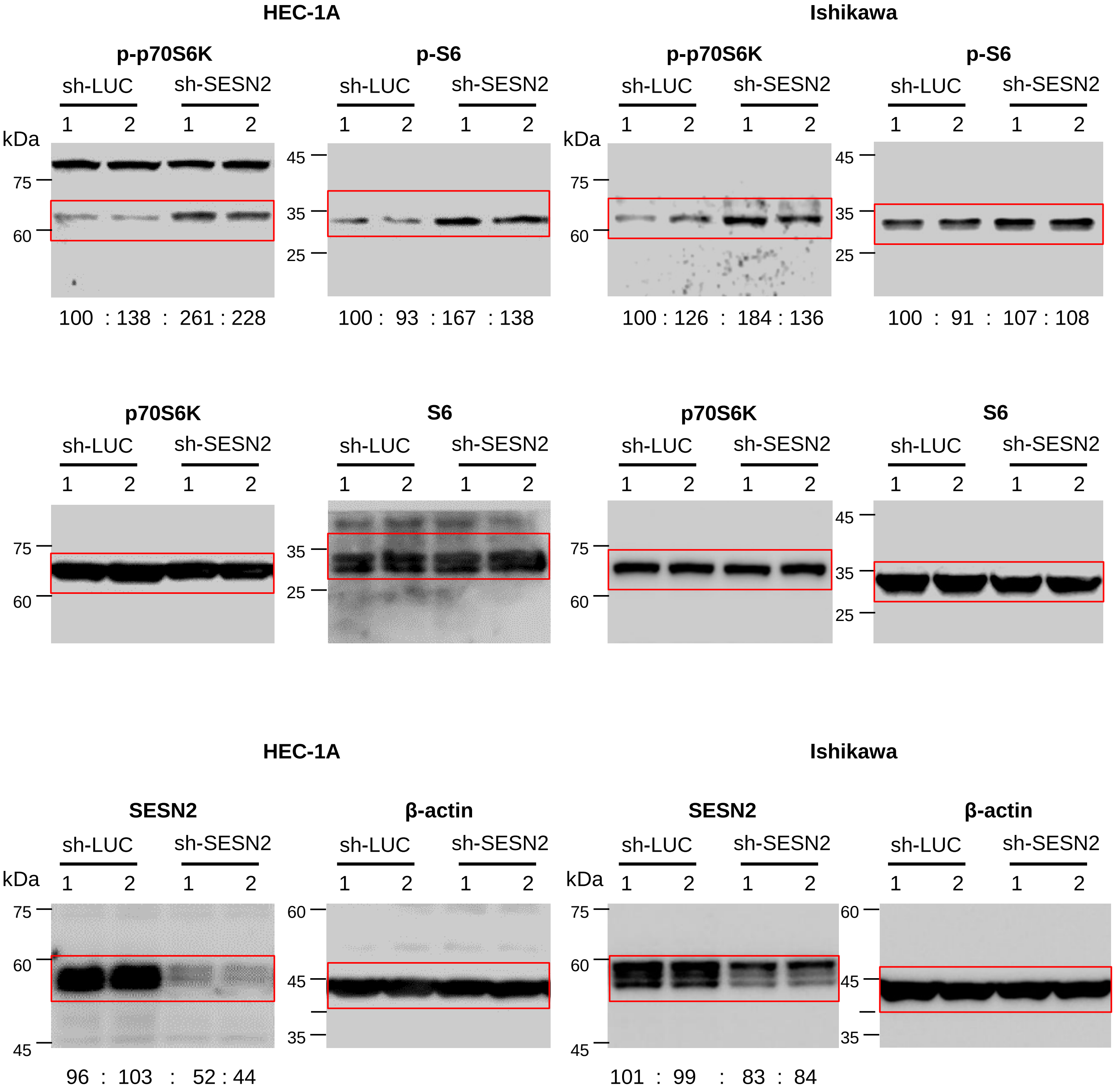
HEC-1A
Ishikawa
p-p70S6K
p-S6
sh-LUC
sh-SESN2
1 2 1 2
kDa
75
60
45
35
25
100 : 138 : 261 : 228
100 : 93 : 167 : 138
100 : 126 : 184 : 136
100 : 91 : 107 : 108
p70S6K
S6
SESN2
β-actin
96 : 103 : 52 : 44
101 : 99 : 83 : 84

Figure 4C

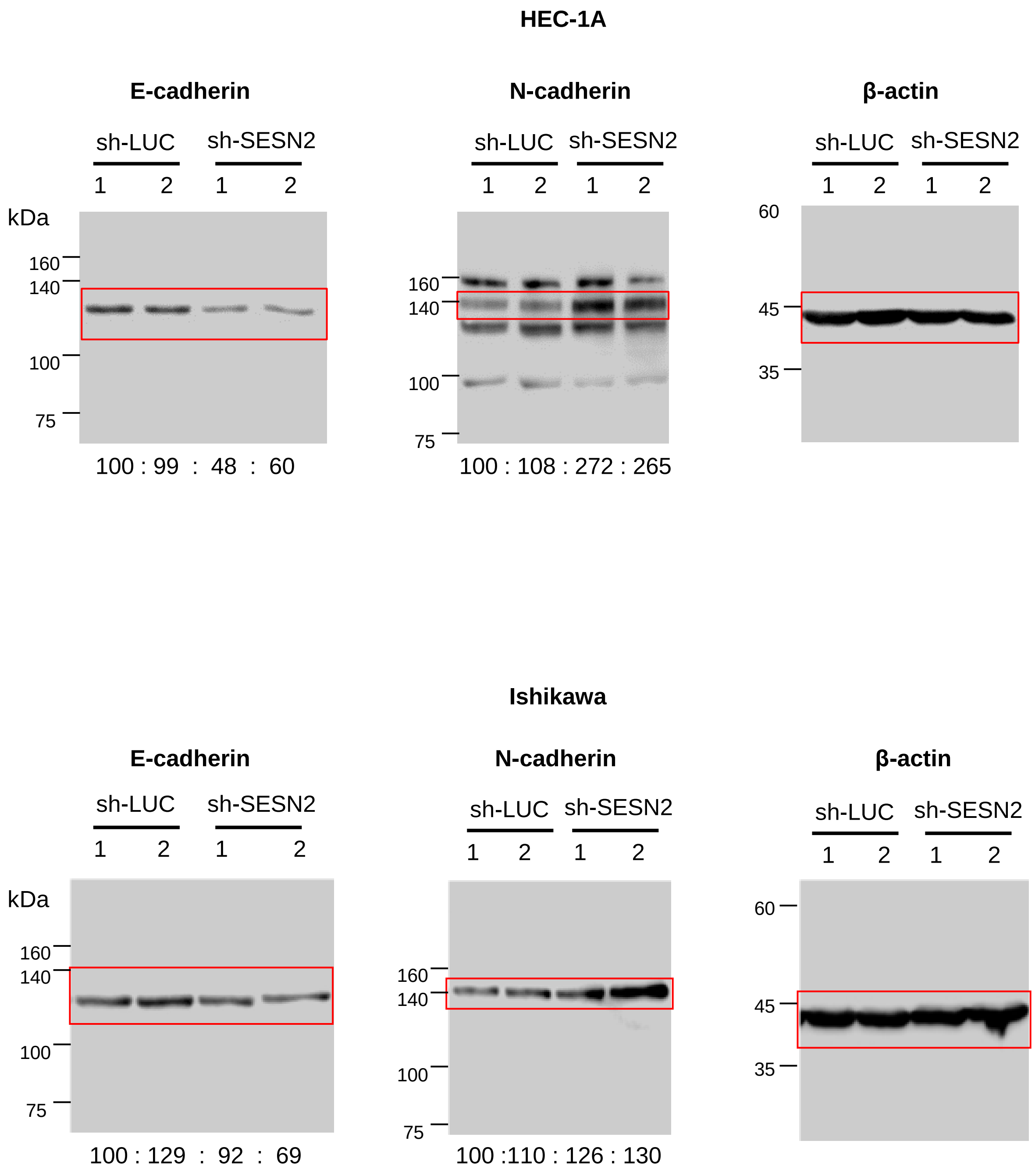

HEC-1A
E-cadherin
N-cadherin
β-actin
sh-LUC
sh-SESN2
1 2 1 2
kDa
160
140
100
75
60
45
35
100 : 99 : 48 : 60
100 : 108 : 272 : 265
Ishikawa
E-cadherin
N-cadherin
β-actin
sh-LUC
sh-SESN2
1 2 1 2
kDa
160
140
100
75
60
45
35
100 : 129 : 92 : 69
100 :110 : 126 : 130

Figure 5A

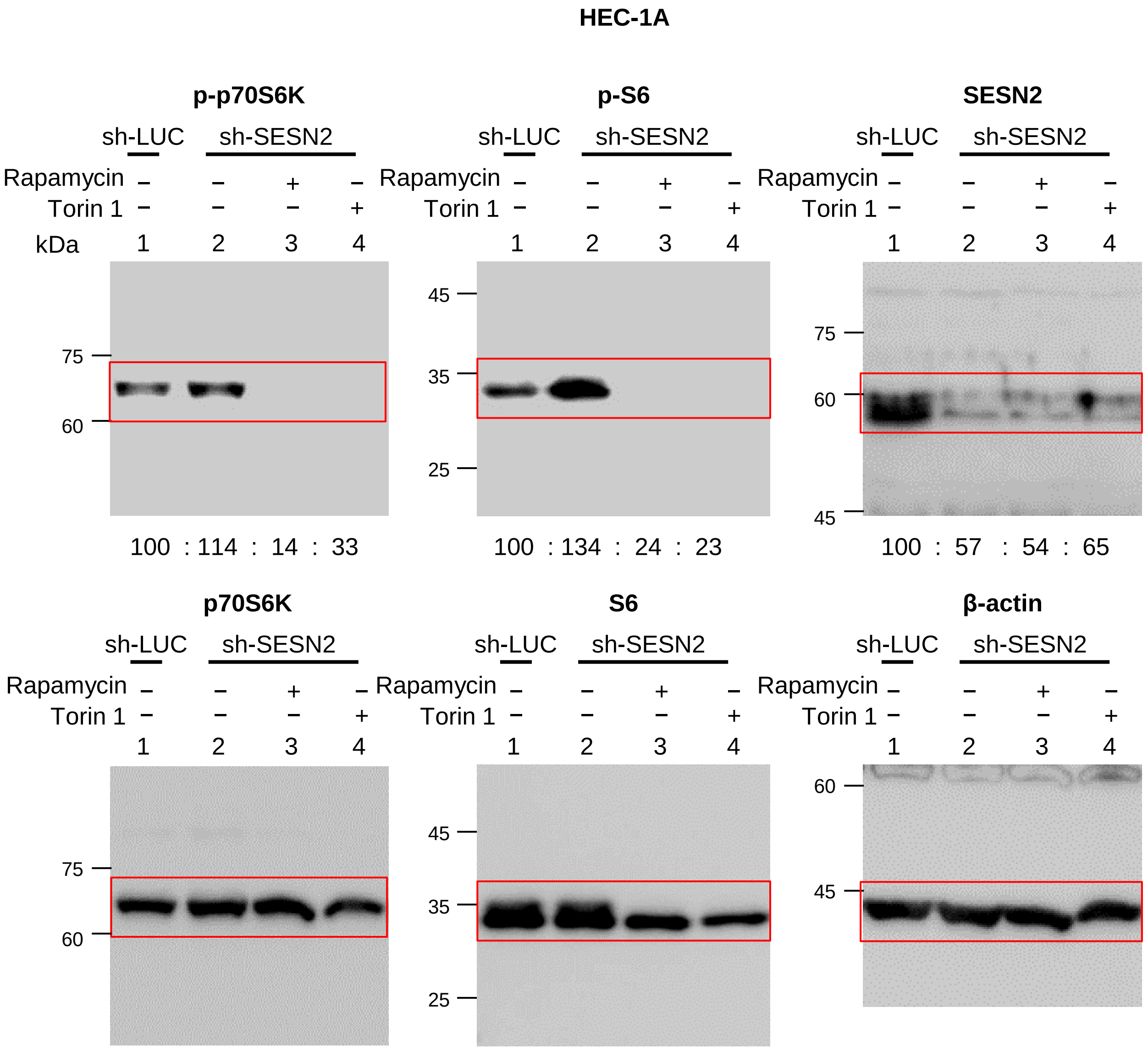
HEC-1A
p-p70S6K
p-S6
SESN2
sh-LUC
sh-SESN2
Rapamycin
Torin 1
kDa
1 2 3 4
75
60
45
35
25
100 : 114 : 14 : 33
100 : 134 : 24 : 23
100 : 57 : 54 : 65
p70S6K
S6
β-actin

Figure 5A

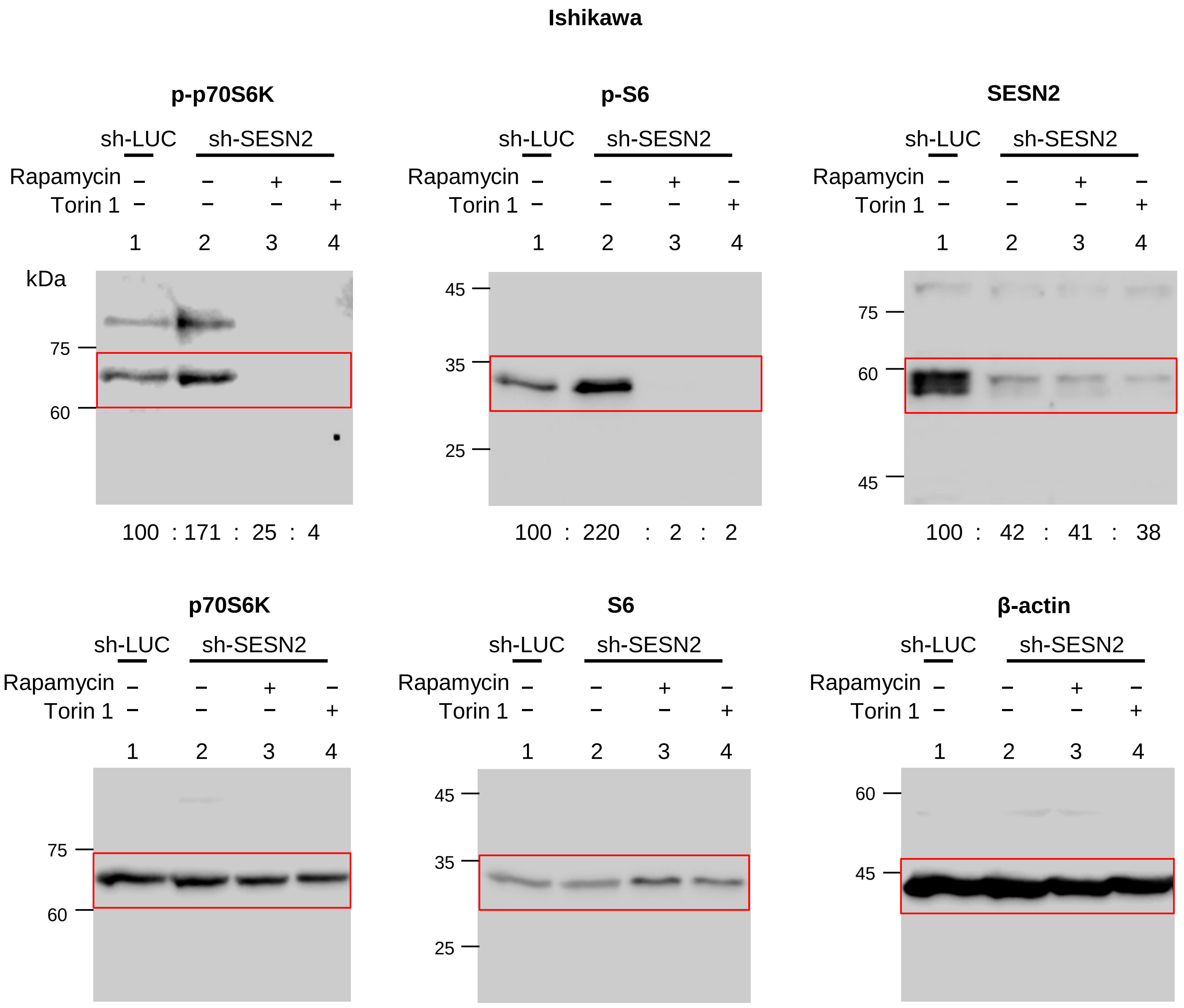
Ishikawa
p-p70S6K
sh-LUC sh-SESN2
Rapamycin – – + –
Torin 1 – – – +
1 2 3 4
kDa
75
60
100 : 171 : 25 : 4
p-S6
sh-LUC sh-SESN2
Rapamycin – – + –
Torin 1 – – – +
1 2 3 4
45
35
25
100 : 220 : 2 : 2
SESN2
sh-LUC sh-SESN2
Rapamycin – – + –
Torin 1 – – – +
1 2 3 4
75
60
45
100 : 42 : 41 : 38
p70S6K
sh-LUC sh-SESN2
Rapamycin – – + –
Torin 1 – – – +
1 2 3 4
75
60
S6
sh-LUC sh-SESN2
Rapamycin – – + –
Torin 1 – – – +
1 2 3 4
45
35
25
β-actin
sh-LUC sh-SESN2
Rapamycin – – + –
Torin 1 – – – +
1 2 3 4
60
45

Figure 7A

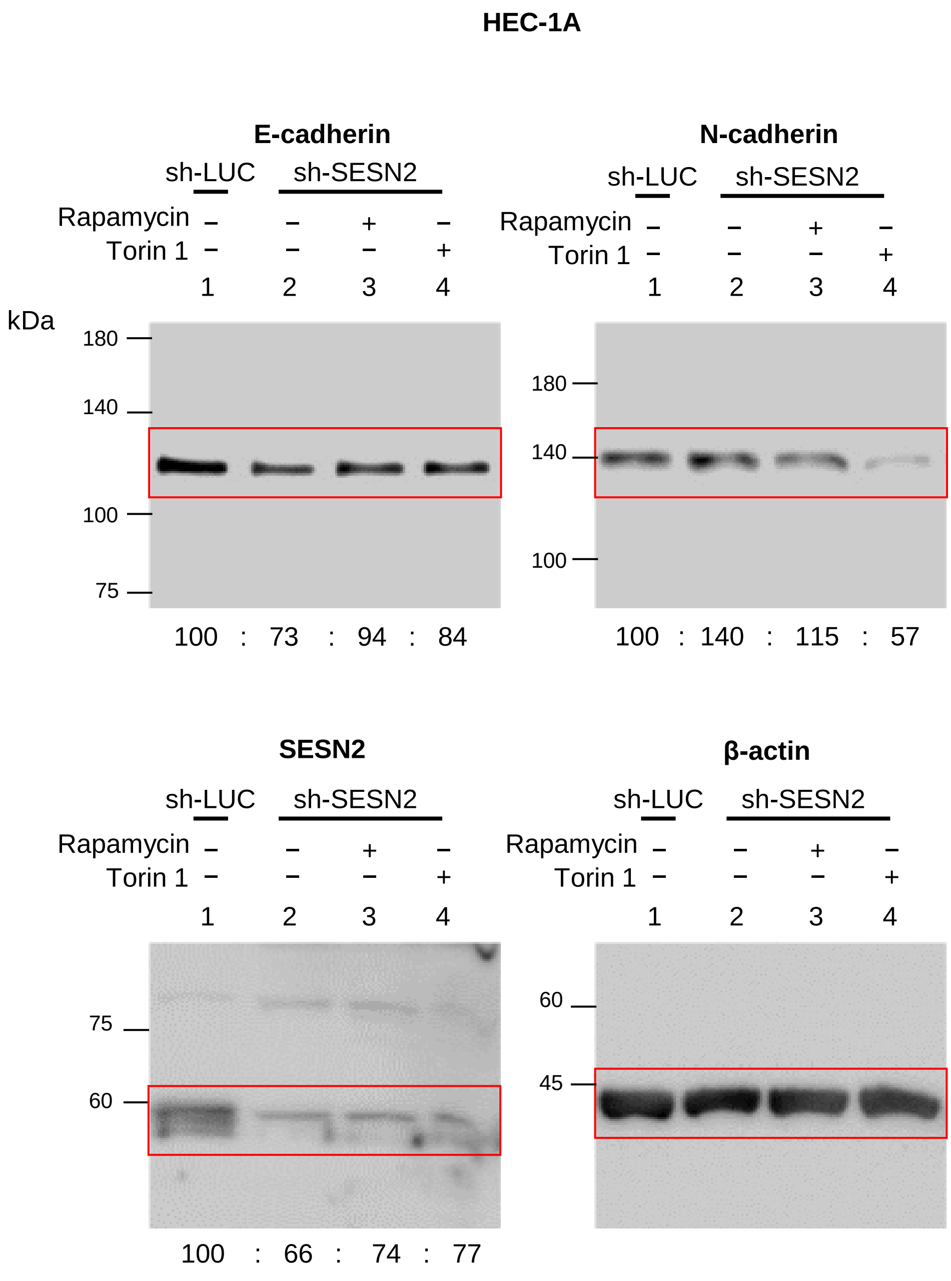
HEC-1A
E-cadherin
sh-LUC
sh-SESN2
Rapamycin – – + –
Torin 1 – – – +
1 2 3 4
kDa
180
140
100
75
100 : 73 : 94 : 84
N-cadherin
sh-LUC
sh-SESN2
Rapamycin – – + –
Torin 1 – – – +
1 2 3 4
180
140
100
100 : 140 : 115 : 57
SESN2
sh-LUC
sh-SESN2
Rapamycin – – + –
Torin 1 – – – +
1 2 3 4
75
60
100 : 66 : 74 : 77
β-actin
sh-LUC
sh-SESN2
Rapamycin – – + –
Torin 1 – – – +
1 2 3 4
60
45

Figure 7A

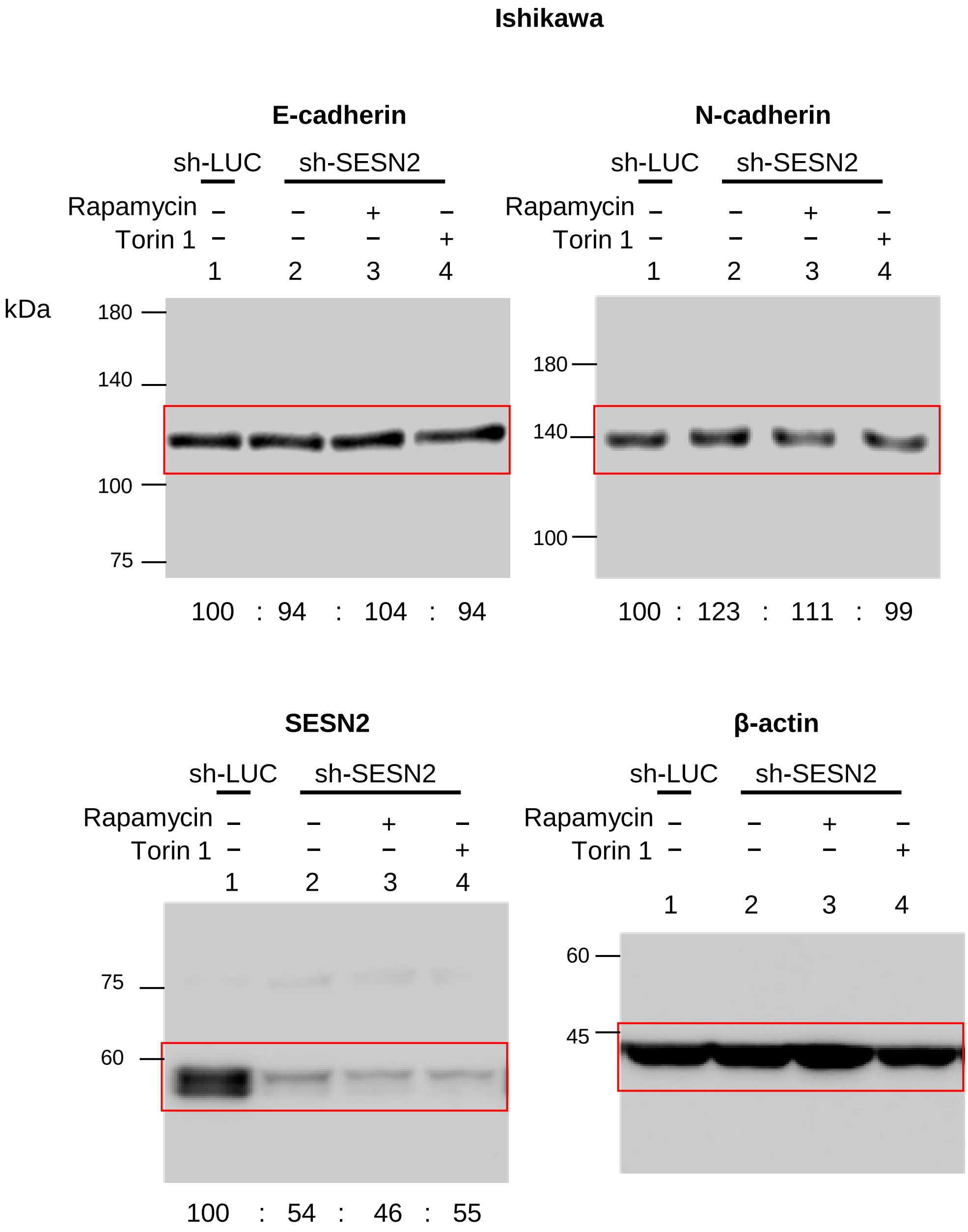
Ishikawa
E-cadherin
sh-LUC
sh-SESN2
Rapamycin – – + –
Torin 1 – – – +
1 2 3 4
kDa
180
140
100
75
100 : 94 : 104 : 94
N-cadherin
sh-LUC
sh-SESN2
Rapamycin – – + –
Torin 1 – – – +
1 2 3 4
180
140
100
100 : 123 : 111 : 99
SESN2
sh-LUC
sh-SESN2
Rapamycin – – + –
Torin 1 – – – +
1 2 3 4
75
60
100 : 54 : 46 : 55
β-actin
sh-LUC
sh-SESN2
Rapamycin – – + –
Torin 1 – – – +
1 2 3 4
60
45

Figure 7B

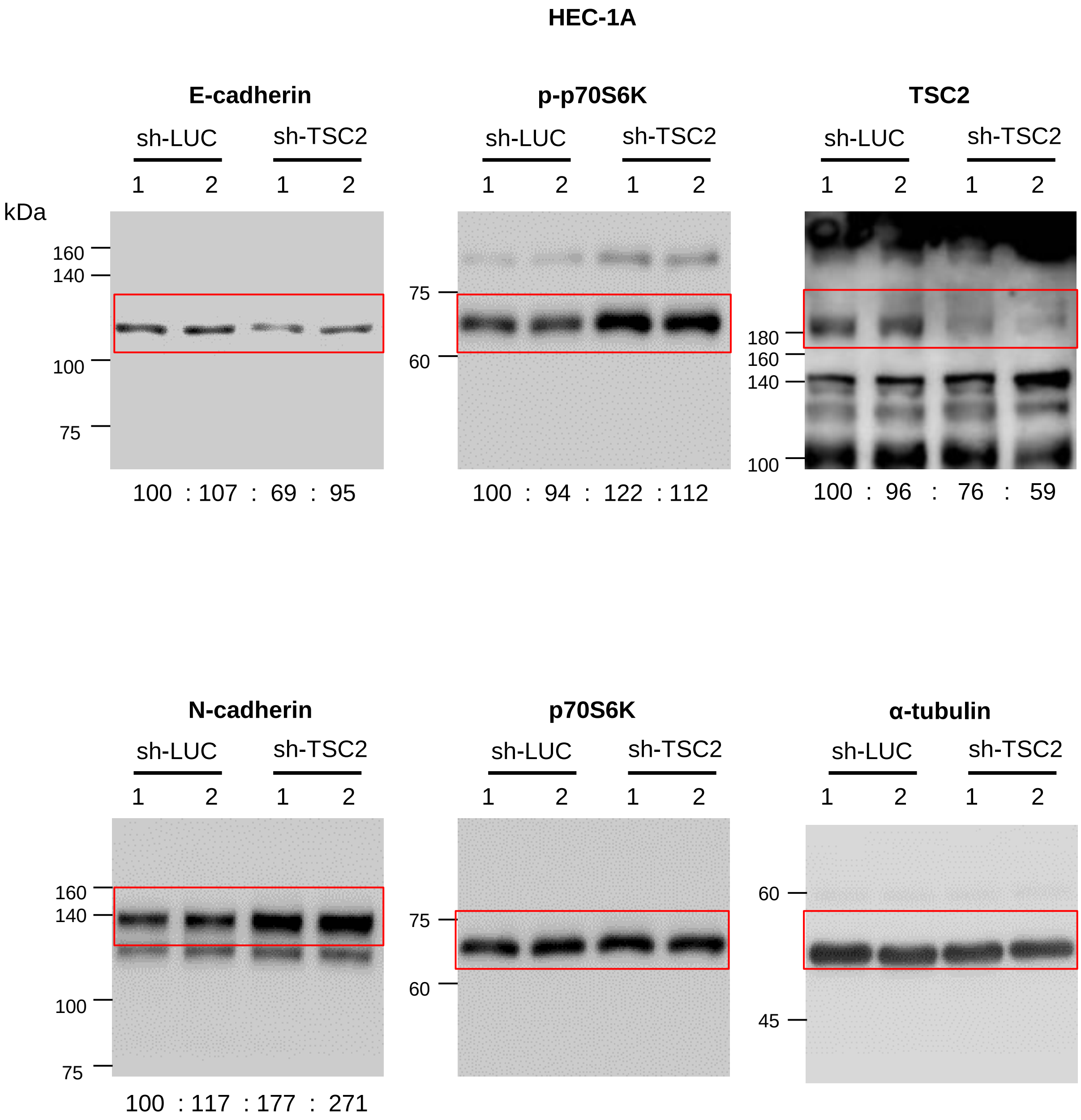
HEC-1A
E-cadherin
p-p70S6K
TSC2
sh-LUC
sh-TSC2
1 2 1 2
kDa
160
140
100
75
60
180
100 : 107 : 69 : 95
100 : 94 : 122 : 112
100 : 96 : 76 : 59
N-cadherin
p70S6K
α-tubulin
45
100 : 117 : 177 : 271

:

Figure 7B

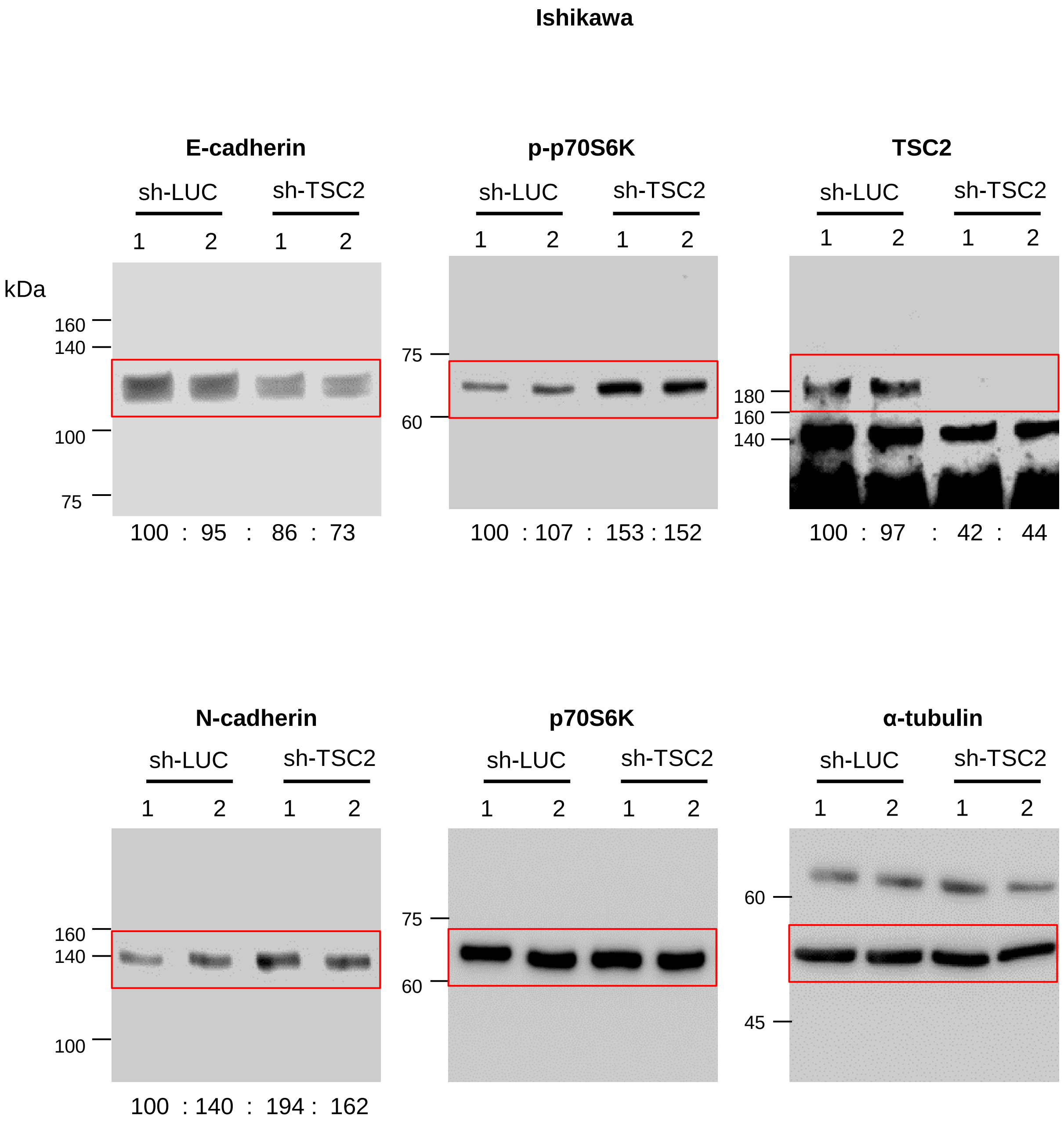

Ishikawa
E-cadherin
p-p70S6K
TSC2
sh-LUC
sh-TSC2
1 2 1 2
kDa
160
140
100
75
60
180
100 : 95 : 86 : 73
100 : 107 : 153 : 152
100 : 97 : 42 : 44
N-cadherin
p70S6K
α-tubulin
45
100 : 140 : 194 : 162

Figure 7C

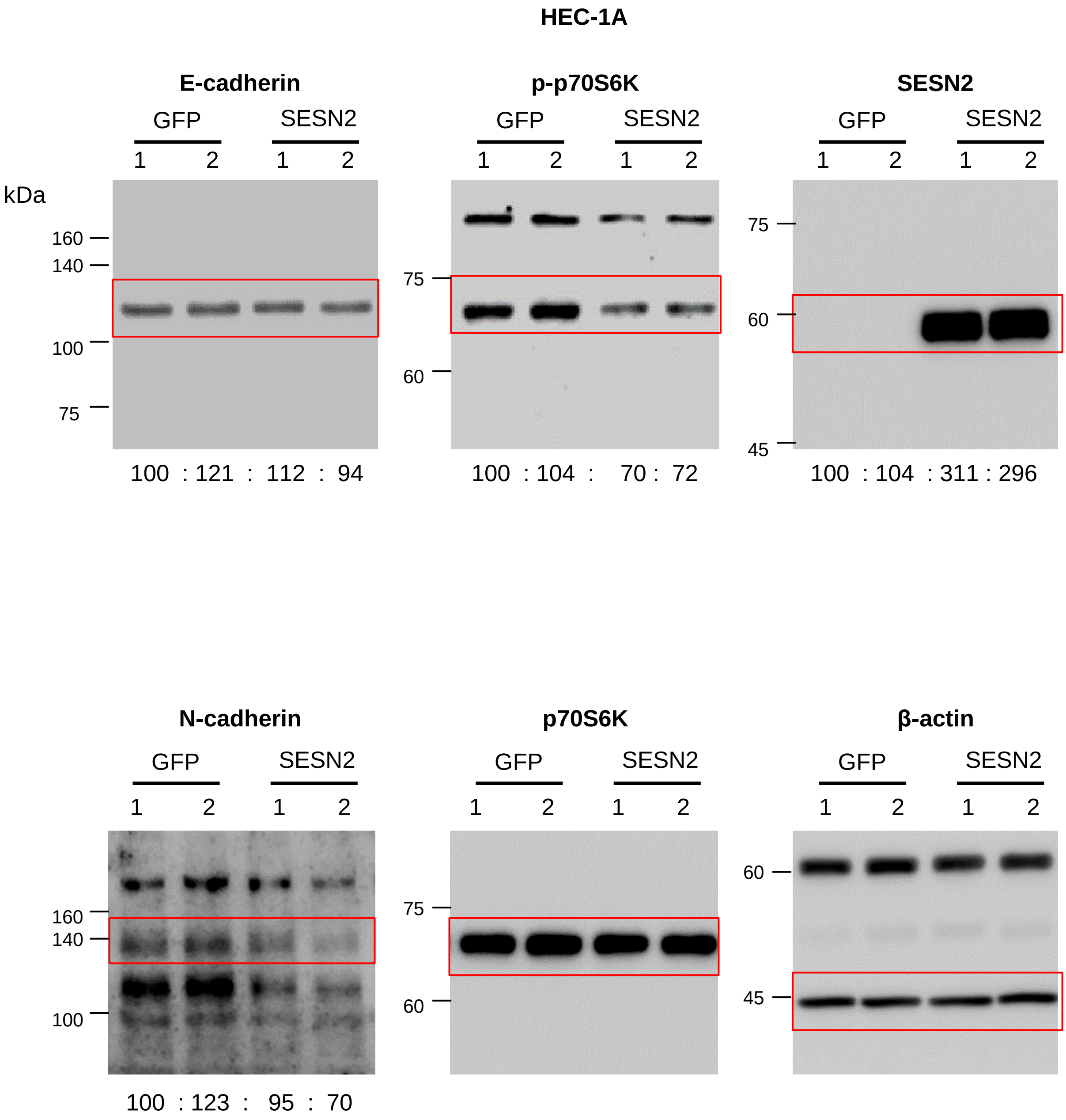
HEC-1A
E-cadherin
GFP
SESN2
1 2 1 2
kDa
160
140
100
75
100 : 121 : 112 : 94
p-p70S6K
75
60
100 : 104 : 70 : 72
SESN2
75
60
45
100 : 104 : 311 : 296
N-cadherin
160
140
100
100 : 123 : 95 : 70
p70S6K
75
60
β-actin
60
45

Figure 7C

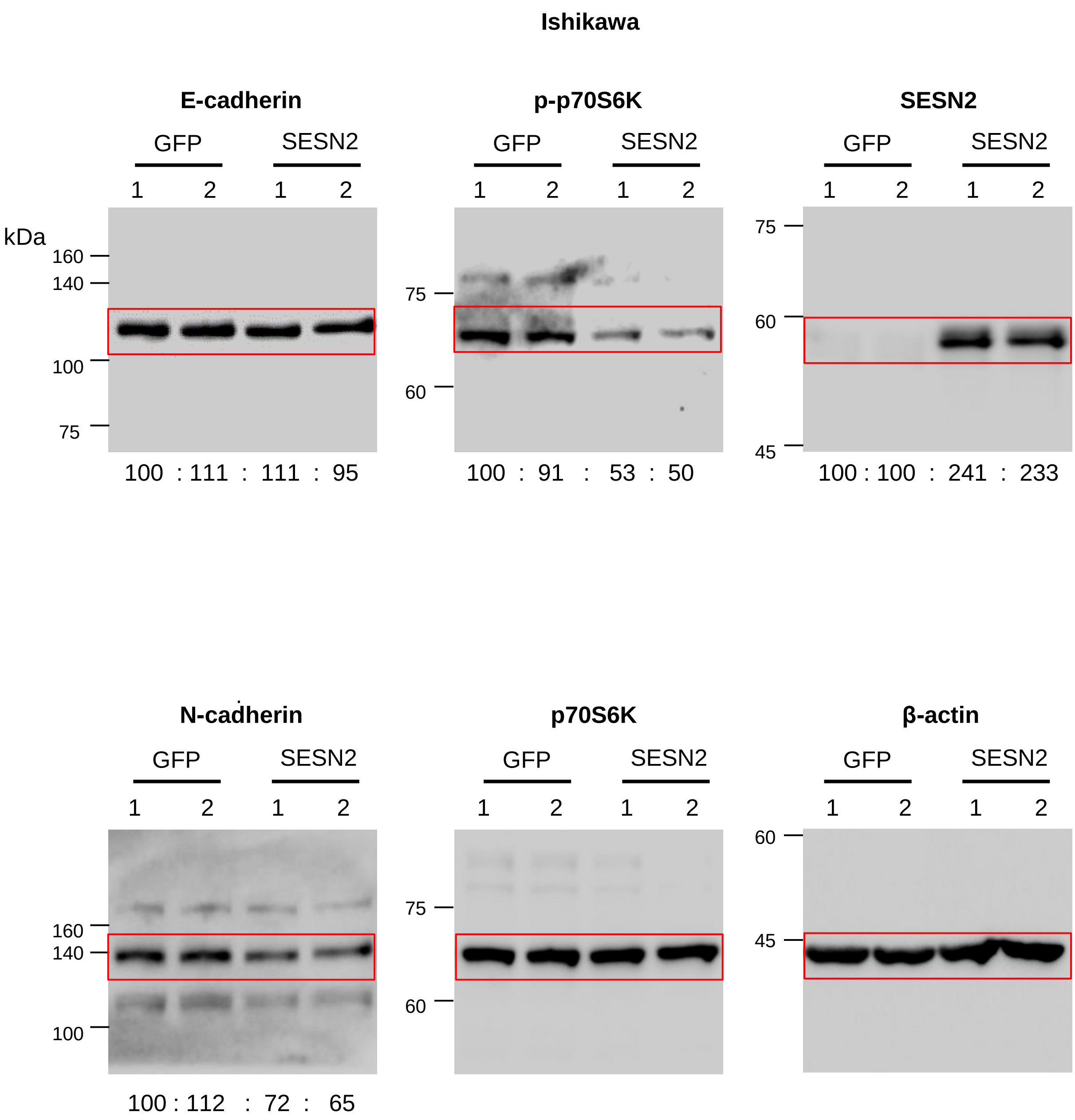
Ishikawa
E-cadherin
GFP
SESN2
1 2 1 2
kDa
160
140
100
75
100 : 111 : 111 : 95
p-p70S6K
GFP
SESN2
1 2 1 2
75
60
100 : 91 : 53 : 50
SESN2
GFP
SESN2
1 2 1 2
75
60
45
100 : 100 : 241 : 233
N-cadherin
GFP
SESN2
1 2 1 2
160
140
100
100 : 112 : 72 : 65
p70S6K
GFP
SESN2
1 2 1 2
75
60
β-actin
GFP
SESN2
1 2 1 2
60
45

Figure 8E

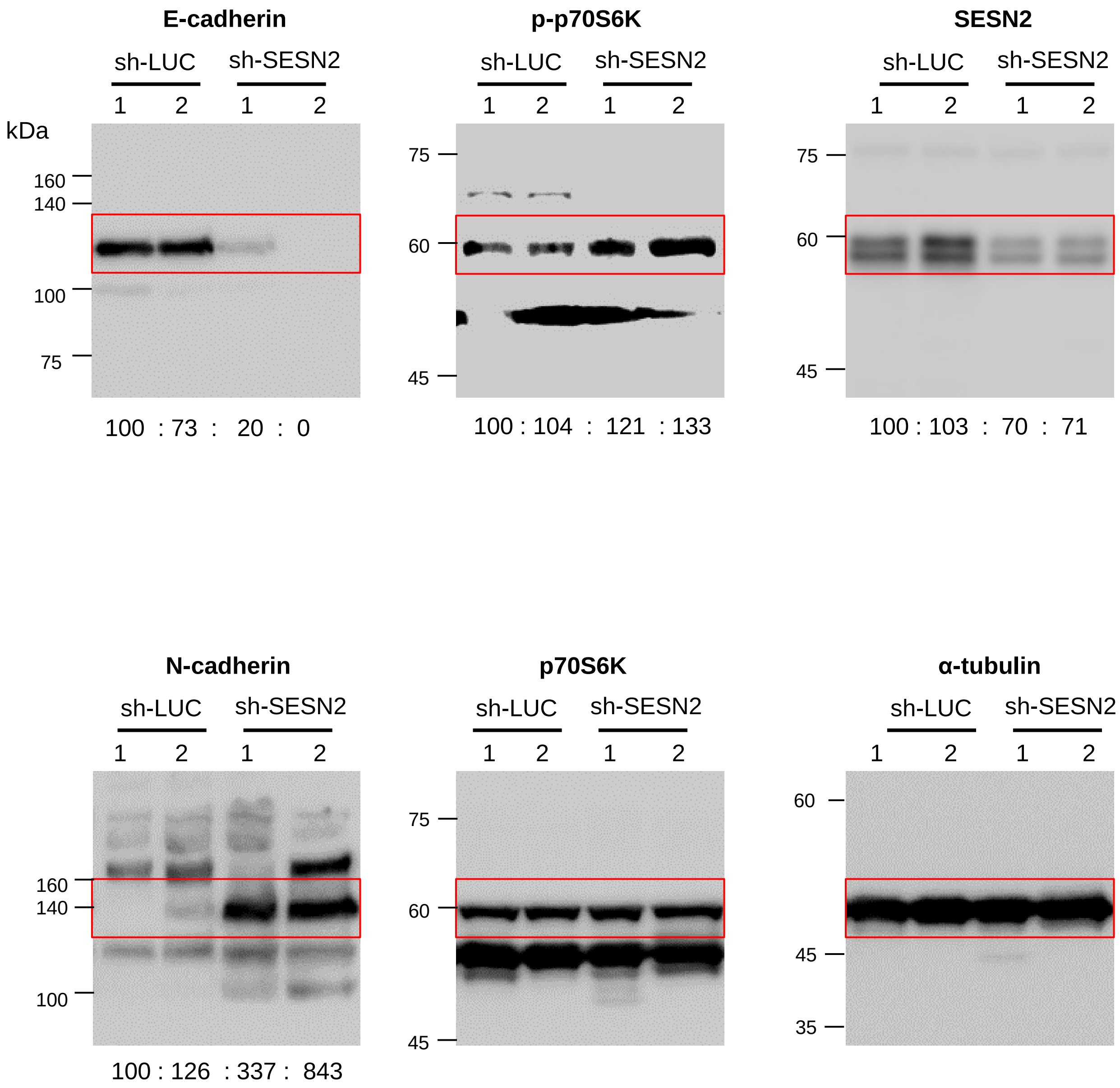
E-cadherin
sh-LUC
sh-SESN2
1 2 1 2
kDa
160
140
100
75
100 : 73 : 20 : 0
p-p70S6K
sh-LUC
sh-SESN2
1 2 1 2
75
60
45
100 : 104 : 121 : 133
SESN2
sh-LUC
sh-SESN2
1 2 1 2
75
60
45
100 : 103 : 70 : 71
N-cadherin
sh-LUC
sh-SESN2
1 2 1 2
160
140
100
100 : 126 : 337 : 843
p70S6K
sh-LUC
sh-SESN2
1 2 1 2
75
60
45
α-tubulin
sh-LUC
sh-SESN2
1 2 1 2
60
45
35

Supplemental Figure 1

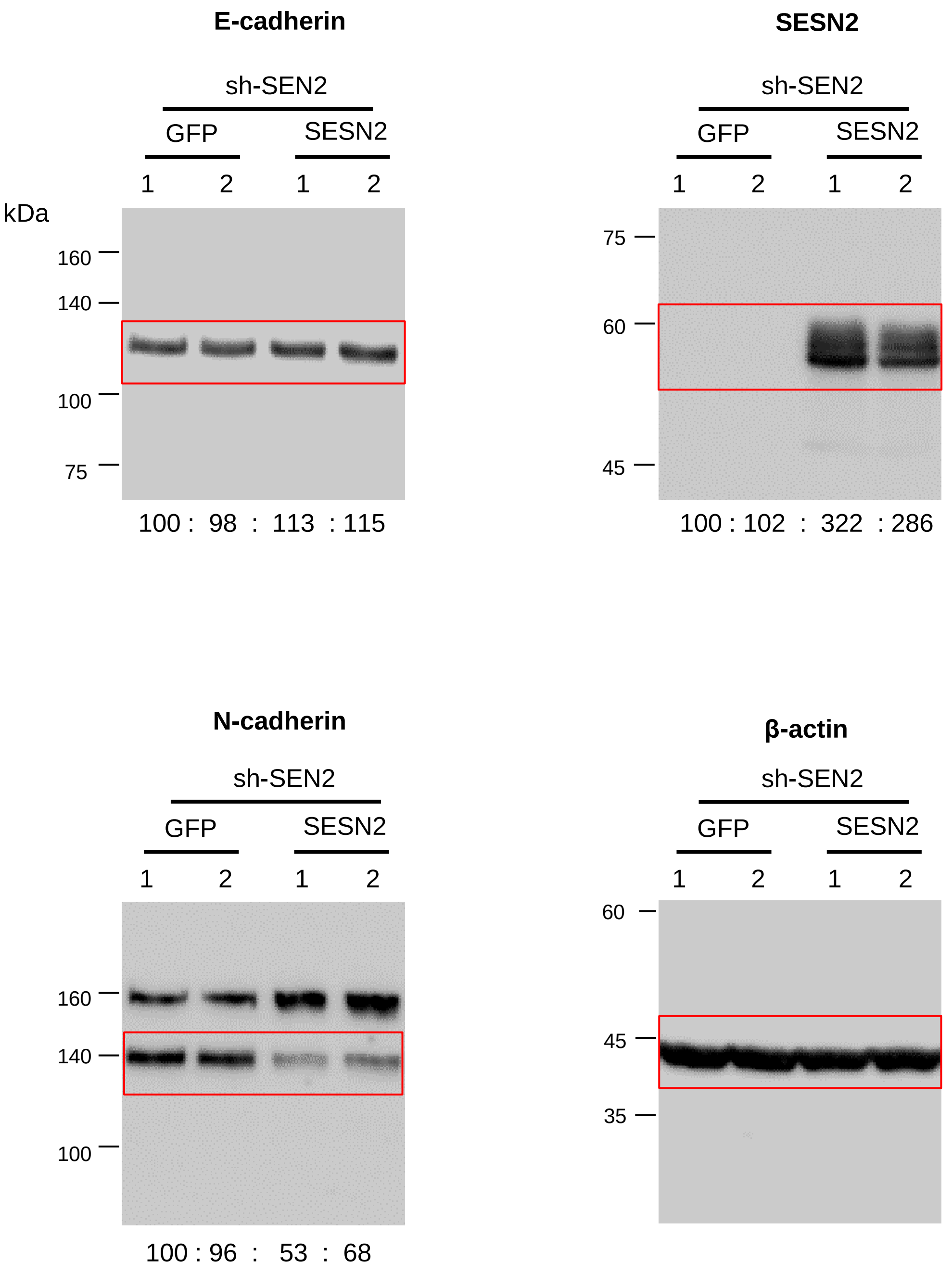

Supplement: Supplementary file 1 [file cancers-12-02515-s001.zip › Cancers 2020_Shin et al_SI-Whole blots R1.pdf]
