# Supplementary material for: mTOR-Dependent Role of Sestrin2 in Regulating Tumor Progression of Human Endometrial Cancer
Source: Cancers (Basel). 2020 Sep 4;12(9):2515. doi: 10.3390/cancers12092515 (PMC7565818; doi:10.3390/cancers12092515)
Supplement: Supplementary file 1 [file cancers-12-02515-s001.zip › Cancers 2020_Shin et al_SI.docx]

Supplementary Materials: mTOR-dependent role of Sestrin2 in regulating tumor progression of human endometrial cancer

Jiha Shin, Jeongyun Bae, Sumi Park, Hyun-Goo Kang, Seong Min Shin, Gunho Won, Jong-Seok Kim, Ssang-Goo Cho, Youngsok Choi, Sang-Muk Oh, Jongdae Shin, Jeong Sig Kim and Hwan-Woo Park


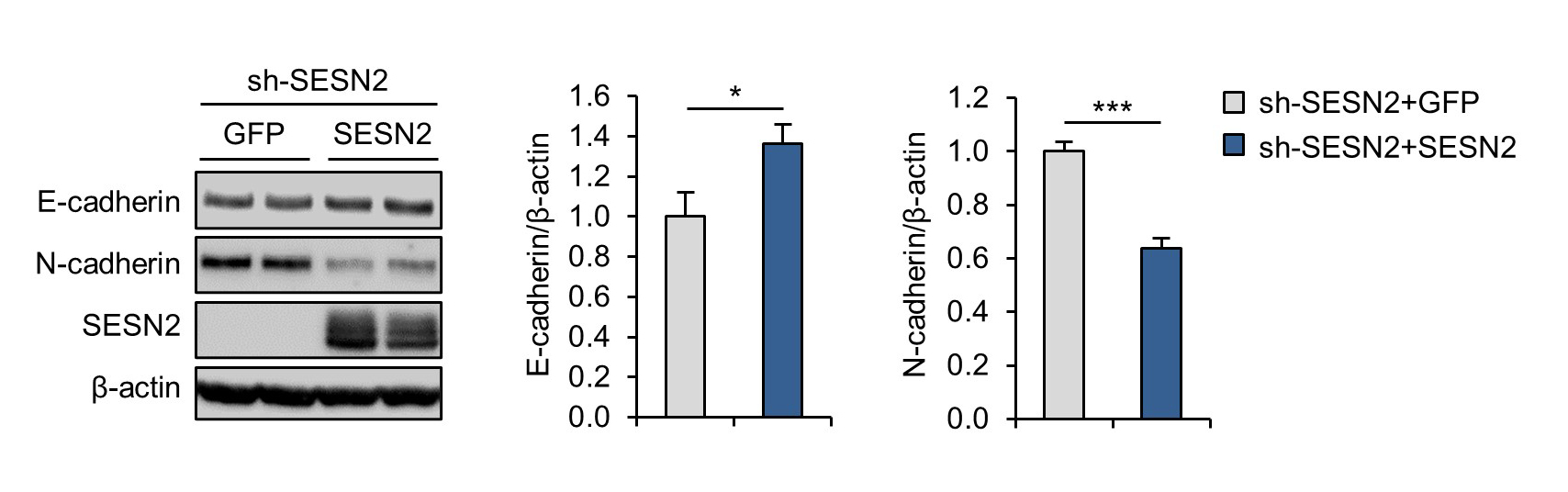


**Figure S1.** Effect of *SESN2* overexpression on EMT in HEC-1A cells with *SESN2* knockdown. Immunoblotting analysis for E-cadherin, N-cadherin, and SESN2 has been performed in *SESN2* knockdown HEC-1A cells infected with lentiviruses expressing GFP as control or overexpressing *SESN2*. β-Actin served as a loading control. Band intensities are quantified and normalized with β-actin values. Data are shown as mean ± SEM. Results are representative of at least three independent experiments. **p* < 0.05; ****p* < 0.001 (Student’s *t*-test).
